# Increased variability in *Apc*^*Min*^/+ intestinal tissue can be measured with microultrasound

**DOI:** 10.1038/srep29570

**Published:** 2016-07-13

**Authors:** A. Fatehullah, S. Sharma, I. P. Newton, A. J. Langlands, H. Lay, S. A. Nelson, R. K. McMahon, N. McIlvenny, P. L. Appleton, S. Cochran, I. S. Näthke

**Affiliations:** 1 Division of Cell & Developmental Biology, College of Life Sciences, University of Dundee, Dundee DD1 5EH, Scotland, UK; 2 Institute for Medical Science and Technology, Medical Research Institute, Ninewells Hospital and Medical School, Dundee DD1 9SY, UK.

## Abstract

Altered tissue structure is a feature of many disease states and is usually measured by microscopic methods, limiting analysis to small areas. Means to rapidly and quantitatively measure the structure and organisation of large tissue areas would represent a major advance not just for research but also in the clinic. Here, changes in tissue organisation that result from heterozygosity in *Apc*, a precancerous situation, are comprehensively measured using microultrasound and three-dimensional high-resolution microscopy. Despite its normal appearance in conventionally examined cross-sections, both approaches revealed a significant increase in the variability of tissue organisation in *Apc* heterozygous tissue. These changes preceded the formation of aberrant crypt foci or adenoma. Measuring these premalignant changes using microultrasound provides a potential means to detect microscopically abnormal regions in large tissue samples, independent of visual examination or biopsies. Not only does this provide a powerful tool for studying tissue structure in experimental settings, the ability to detect and monitor tissue changes by microultrasound could be developed into a powerful adjunct to screening endoscopy in the clinic.

Measuring detailed changes in living tissue currently relies on optical methods that can only report on a relatively small number of cells or areas. Similarly, cancer screening relies on detecting changes in tissue organisation using visual or radiological examination and is restricted to lesions at least several millimetres in size. Conclusive diagnosis of disease is usually achieved by histopathological examination and involves microscopic inspection of sectioned material removed as biopsies or from surgical resections that can report on local cellular changes. Measuring and detecting tissue changes invisible by current methods in larger samples would not only improve cancer screening, but also aid our ability to relate cell biological changes to tissue changes more readily in the research setting. Identifying methods that can report on subtle, abnormal tissue changes requires proof of concept studies for linking detailed optical data that are known to represent pre-malignancy to more immediately quantitative techniques.

The molecular changes that underpin some cancers are well understood and provide extremely useful models to identify tissue changes in early transformation. In colorectal cancer, inactivation of a single gene, the adenomatous polyposis coli (*Apc*) tumour suppressor, is common to more than 90% of tumours and these mutations occur very early in tumourigenesis (https://www.oncomine.org). Furthermore, heterozygosity in *Apc* is a precancerous condition. *Apc*^*Min*/+^ mice are heterozygous for a truncating *Apc* mutation at codon 850 and invariably develop numerous intestinal and some colonic adenomas^1^,^2^,^3^. This mouse model mimics Familial Adenomatous Polyposis (FAP) in humans who are heterozygous for truncation mutations in *Apc* and present with numerous (100’s to 1,000’s) polyps that progress to cancer if left untreated^4^. In both cases, polyps and tumours have lost or carry mutations in the remaining wild type *Apc* allele. Polyps and tumours are easily recognisable by irregular crypt structure and cellular packing. However, before the appearance of aberrant crypt foci, polyps or adenoma; and distant from these structures, *Apc* heterozygous tissue appears ‘histologically normal’, which we define as indistinguishable from wild type tissue when visualised in two-dimensional sections (i.e. by conventional pathological approaches)^5^,^6^,^7^,^8^.

The high penetrance of *Apc* mutations relates to the multi-functionality of the APC protein. APC contributes directly and indirectly to all the cellular processes that govern normal maintenance of intestinal and colonic epithelia^9^. Heterozygosity of *Apc* influences many processes that affect tissue organisation and structure including apoptosis^10^, Notch signalling^11^, altered differentiation^12^ and proliferation. Multiple studies have reported proteomic and genomic changes in tissue from patients and mouse models carrying heterozygous mutations in *Apc*. However, high-resolution and/or quantitative comparisons of normal tissue distant from polyps or tumours and lacking aberrant crypt foci is not available from FAP patients or *Apc*^*Min*/+^ mice. Since altered tissue architecture is a hallmark of transformation, we aimed to quantitate changes induced by heterozygous mutations in *Apc* before any visible signs of tissue abnormalities occurred. We used microultrasound (μUS) and high-resolution two-photon fluorescence microscopy to quantitatively compare architectural tissue features in three dimensions. We compared histologically normal tissue, using proximal, medial, and distal regions of the small (R1, R2, R3) and large (R4, R5, R6) intestine (see Suppl. Figure S1 for schematic representation of the positions of these regions along the intestinal axis) in wild type (WT) and *Apc*^*Min*/+^ mice^1^,^2^ and in tissue from one normal and one FAP patient.

We found that backscatter (BSC) and acoustic impedance (Z), measured by microultrasound (μUS), were both increased and more variable in *Apc* heterozygous tissue. Microultrasound could also reliably detect overt signs of tissue changes in minute polyps. High-resolution, three-dimensional microscopic measurements revealed that the organisation of *Apc* heterozygous tissue is more variable in mice and humans: crypts were more curved and they were more irregularly packed than in corresponding wild type tissue. Together these data suggest that more irregular tissue organisation is a feature of precancerous tissue and can be detected by microultrasound. Microultrasound is an immediately quantitative, high-resolution technique that can act as a powerful tool in tissue biology. It also has the potential to be combined with endoscopic imaging for screening of the intestinal tract. Our observations act as proof of principle for the potential utility of μUS for monitoring aberrant tissue organisation in development and disease.

## Results

### Microultrasound detects altered properties of precancerous tissue

**Figure 1:**
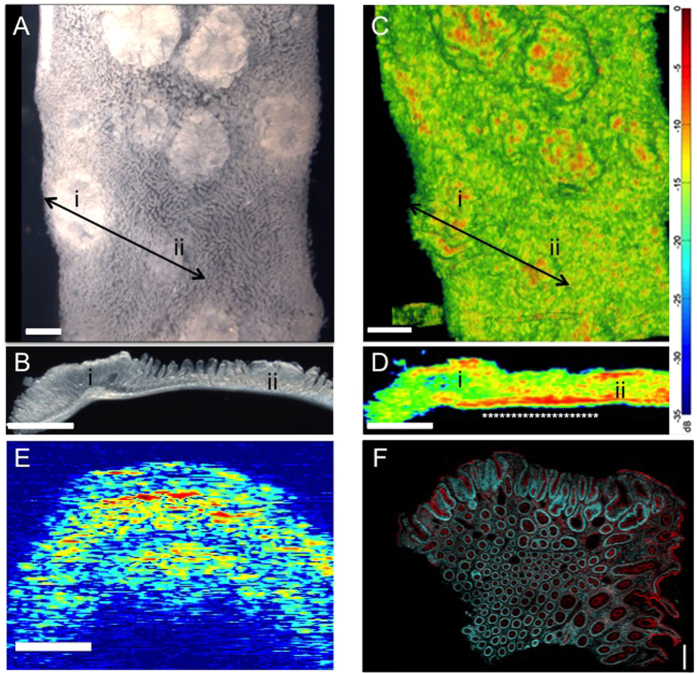
Microultrasound scanning of intestine detects altered tissue organisation. (**A**) Intestinal tissue from *Apc*^*Min/+*^ mice was pinned onto agar and viewed at 10X magnification revealing superficial polyps. The arrow connecting the polyps visible at ‘i’ and ‘ii’ shows the region of polyp that was resected and sectioned with a vibratome after visual and ultrasound examination to allow visual examination in cross section. (**B**) Bright field image of the cross section indicated by the line between i and ii in panel A. (**C**) 3-D composite image generated from 580 individual 45 MHz ultrasound B-mode scans obtained by scanning over the same tissue sample as in (**A**). The polyps appear as hyper-echoic ‘hot’ spots when mapped to a relative heat map using logarithmic compression. The dynamic range notation used remains unchanged, with the largest signal amplitude mapped to 1 corresponding to 0 dB and −35dB to the smallest signal. (See colour bar on right). An example of a single B-scan showing the unprocessed signal is shown in Suppl. Figure S2. (**D**) Ultrasound B-scan along the cross-section labeled in (**C**) using the same colour map. Polyps marked ‘i’ and ‘ii’ are visible as areas of high reflectivity localized superficially. High intensity signal on the underside of the tissue corresponds to the muscle layer (white asterisks). (**E**) Unprocessed μUS scan of the middle of a human polyp from FAP patient and (**F**) corresponding image of a cross section cut from the same polyp and stained with phalloidin and DAPI to reveal F-actin (red) and nuclei (blue). The overall structure of the polyp is well reported by μUS. (All scale bars = 1 mm).

Precancerous *Apc* heterozygous tissue appears indistinguishable from wild type tissue when visualised by conventional pathological approaches^5^,^6^,^7^,^8^. Detection of subtle changes in tissue organisation that could be indicative of a precancerous state requires quantitative methods for measuring tissue properties at high resolution. μUS is a tool with the potential to detect such changes. To determine if μUS could detect tissue abnormalities, we first established whether differences expected in polyps could be detected. Scanning tissue from *Apc*^*Min*/+^ mice revealed polyps and tumours (Fig. 1A–D). Importantly, μUS was able to detect even small, superficial polyps (≤300 μm in diameter) present in these samples (Fig. 1A–D). We also found that the muscle layer that surrounds the intestine had higher ultrasound signal (Suppl. Figure S2). This allowed its clear visualisation and was consistent with the higher mechanical stiffness and density of muscle cells and tissue^13^. It is well established that crypts and cells in polyps are more irregularly arranged, which likely causes the increased Backscatter (BSC) and acoustic impedance (Z) that contribute to their characteristic ultrasound signal (e.g. polyp ‘ii’, Fig. 1A–D)^5^,^6^,^7^,^8^. Using tissue from a human polyp also showed that plotting the signal intensity of μUS in individual B-scans recapitulates the overall layered organisation of a FAP polyp (Fig. 1E,F). These measurements confirmed μUS as a potentially useful tool to characterise overall tissue organisation, and showed that it can detect polyps <300 μm, which is much smaller than those currently detectable by colonoscopy (6–9 mm)^14^. To determine if the quantitative information generated by μUS signals could detect tissue changes preceding polyp formation, we next scanned tissue samples from male and female mice at ages 60, 90 and 120 days.

**Figure 2:**
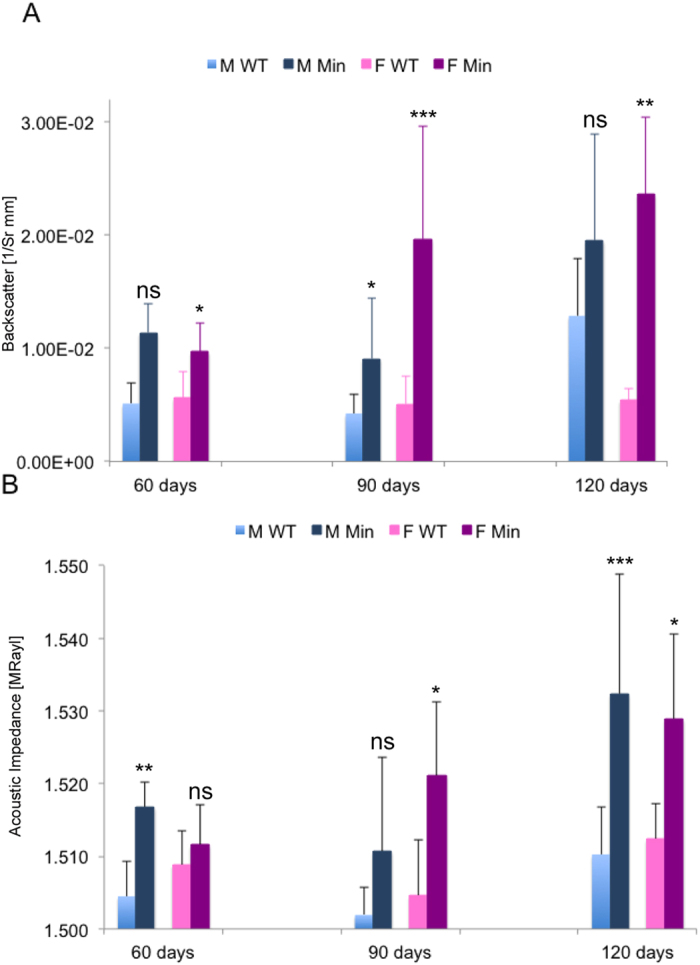
Backscatter and acoustic impedance are increased before tissue changes can be detected visually. WT and *Apc*^*Min/+*^ (Min) tissue specimens from male (M) and female (F) littermates at each of the indicated ages were prepared for μUS scanning and mean (**A**) Backscatter Coefficients (BSC) and (**B**) Acoustic Impedance (*Z*) were determined. Averages with error bars represent data from at least three independent scans each from the following numbers of mice: 60 days: F WT, 3; F Min, 4; M WT, 3; M Min, 4; 90 days: F WT, 6; F Min, 5; M WT, 6; M Min, 5; 120 days: F WT, 6; F Min, 5; M WT, 3; M Min, 6. Statistics were calculated by unpaired t-test with Welch’s correction (assuming samples have unequal variances) ns = not significant, *p < 0.05, **p < 0.01, ***p < 0.001, ****p < 0.0001. Either BSC and/or Z was significantly different at all ages in both genders.

The average acoustic impedance we measured in mouse gut tissue was only slightly lower than recorded values for bovine muscle tissue, consistent with the presence of the softer mucosal layers in our tissue^15^,^16^. Comparing the BSC values we obtained with those previously measured in tissue or simulated tissue ‘phantoms’ is complicated by the fact that most previous studies used different tissue types or cell aggregates and employed ultrasonic frequencies below 30 MHz. The high inter- and intra-tissue variability of this parameter and its dependence on sonication frequency make it difficult to directly compare values we obtained with previous studies^17^,^18^,^19^,^20^,^21^. For this reason our study focused on consistency between samples and a direct comparison between wild type and *Apc*^*Min*/+^ tissue. Indeed, directly comparing data between tissue samples that only varied in the genotype of the animal that they were derived from revealed that BSC, *Z*, or both were significantly elevated and/or more variable in *Apc* heterozygous tissue, even in the absence of abnormalities visible in sectioned, conventionally visualised material (Fig. 2, Suppl. Figure S3, Suppl. Table 1). Increased BSC has been previously demonstrated to be indicative of increased irregularity of tissue structure suggesting that precancerous tissue may be less regularly structured^20^,^22^,^23^.

To determine what caused the increased variance in BSC and Z values in precancerous tissue, we used two-photon laser confocal microscopy to measure tissue architecture in whole tissue at high resolution in three dimensions.

### Crypt packing

**Figure 3:**
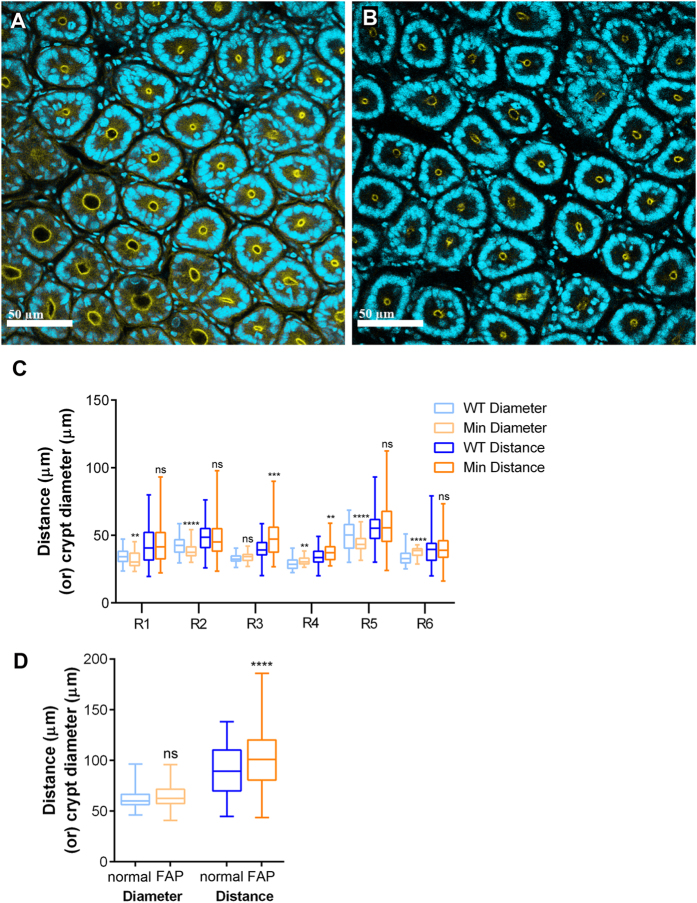
Crypt packing is more variable in *Apc*^*Min/*^+ and FAP tissue. Cross sections of (**A**) WT and (**B**) *Apc*^*Min/+*^ crypts from Region 2 were stained with DAPI to visualise nuclei (cyan) and phalloidin (yellow) to mark F-actin. Scale bars = 50 μm. (**C**) The diameter of crypts (light colours) and the distance between the centre of a crypt and its six nearest neighbouring crypts (darker colours) was measured and is shown in Box plots for each region indicating packing. The ends of each line represent maximum and minimum values. The ‘boxes’ show upper and lower quartile around the median, indicated by the horizontal line. Statistical significance of differences was calculated using unpaired t-test with Welch’s correction. WT = *Apc*^*+/+*^, Min = *Apc*^*Min/+*^ (In each case, the number of crypts (n) that were scored in each region were: for distance: WT, R1 n = 120, R2 n = 180, R3 n = 60, R4 n = 60, R5 n = 180, R6 n = 120; *Apc*^*Min/+*^, R1 n = 120, R2 n = 240, R3 n = 60, R4 n = 60, R5 n = 240, R6 n = 120, and for diameter: WT; R1 n = 100, R2 n = 150, R3 n = 50, R4 n = 50, R5 n = 150, R6 n = 100; *Apc*^*Min/+*^, R1 n = 100, R2 n = 200, R3 n = 50, R4 n = 50, R5 n = 49, R6 n = 50). In each case, tissue images from the following number of mice were used: WT: R1, 2; R2, 3; R3, 1; R4, 1; R5, 3, R6, 2; *Apc*^*Min/+*^: R1, 2; R2, 4; R3, 1; R4, 1; R5, 2; R6, 1. (**D**) Crypt packing and size was measured in human normal and FAP tissue collected from one patient each and displayed as in (**A**). (Number of crypts measured was: distance: WT, n = 240; FAP n = 240; diameter: WT, n = 106, FAP, n = 100). Statistical significance was calculated using unpaired t-test with Welch’s correction: ns = not significant, *p < 0.05, **p < 0.01, ***p < 0.001, ****p < 0.0001. Either mean or variance is significantly different in all regions (except R3). See also Suppl. Tables 2–4.

Intestinal tissue is characterised by regularly arranged crypts that orient perpendicular to the intestinal wall. The relative size and packing density of crypts changes along the proximal – distal axis, but the overall organisation is preserved^24^. To obtain a quantitative measure of how crypts are arranged in the tissue plane and determine if this changes when one copy of *Apc* is lost, we compared crypt packing in six regions along the intestinal and colonic axis in WT and *Apc*^*Min*/+^. Because the size of crypts could affect their packing, we first compared crypt diameters, obtained by measuring the cross section area of crypts (Fig. 3, Suppl. Table 2). The diameter of WT and *Apc*^*Min*/+^ crypts was slightly, but significantly different in all regions except in Region 3 (the terminal 10% of the small intestine) (Fig. 3C, Suppl. Table 2). However, there was no consistent trend in these differences and in some areas WT crypts were larger (regions 1, 2, and 5), while in others they were smaller (Regions 3, 4 and 6) (Suppl. Table 2).

Next we quantified crypt packing by measuring the distances between individual crypts and their six neighbouring crypts. In all regions, except region 6, the maximum distance between crypts in *Apc* heterozygous tissue was higher, resulting in distribution of the distances skewed towards higher values (Fig. 3C, Suppl. Table 3). The variance was significantly higher in regions 1, 2, 3 and 5, and in regions 3 and 4 crypts were significantly further apart (Fig. 3C). Together these data suggest that spacing of crypts in *Apc*^*Min*/+^ tissue is more variable and more irregular.

We compared our observations from mouse with human samples by using tissue corresponding to region 3 (ileum) resected from a FAP patient and from a non-FAP patient, representing FAP and normal respectively. In both cases, tissue was distant from tumours or polyps. There was no significant difference between the space occupied by crypts in FAP and normal tissue as indicated by their similar diameter. The average distance between neighbouring crypts was significantly larger (p < 0.0001) and it was more variable (p = 0.0011) in human FAP tissue (Fig. 3D, Suppl. Table 3). This is also illustrated by the fact that the maximum and minimum distance between neighbouring crypts varied by a factor of 3.1 in normal and 4.2 in FAP tissue. The distribution of the data for inter-crypt distance was also skewed towards higher values in FAP tissue (Suppl. Table 4).

These data suggest that crypts in FAP tissue are further apart and more irregularly spaced than in normal tissue (Fig. 3D, Suppl. Table 4). This is identical to the situation in mouse *Apc*^*Min*/+^ intestine. This difference in crypt packing between normal and *Apc* heterozygous tissue could not be explained by a change in crypt diameter in human FAP tissue, which was not significantly larger than in normal tissue (Fig. 3D, Suppl. Table 4).

### Crypt shape

**Figure 4:**
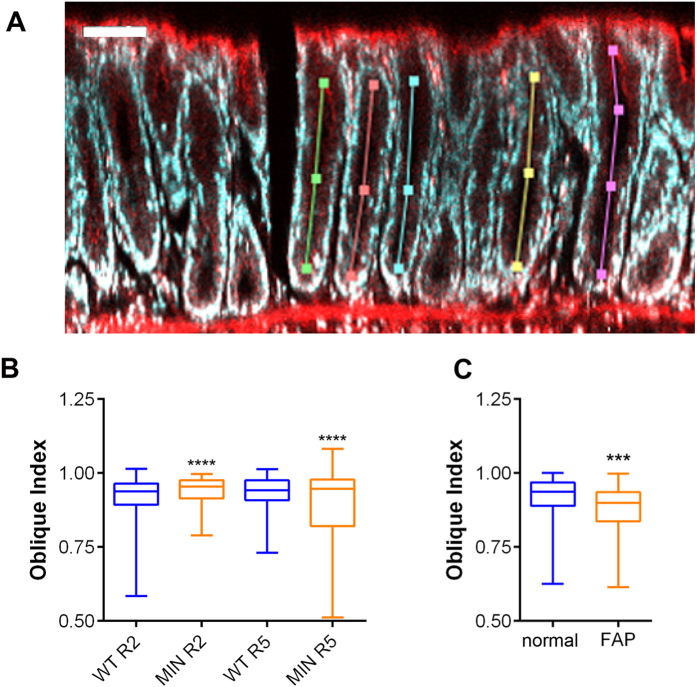
Crypts are more curved in *Apc*^*Min/*^+ and FAP tissue. (**A**) Tissue from region 5 in WT mice stained with DAPI to visualise nuclei (cyan) and rhodamine phalloidin (red) to mark F-actin is shown in cross section revealing the crypt and colonic lumen. Coloured lines follow the path of crypts. Scale bar = 70 μm. (**B**) Crypt curvature (‘oblique index’) was measured as a ratio of crypt depth and length and plotted as Box plots showing crypt curvature in tissue from WT (blue) mice and *Apc*^*Min/+*^ (orange) in Region 2 and 5. A ratio = 1 denotes a straight crypt; ratios <1 denote curved crypts. (Region 2 WT, n = 579, 5 mice, *Apc*^*Min/+*^, n = 649, 2 mice; Region 5 WT, n = 450 crypts, 5 mice; *Apc*^*Min/+*^, n = 349, 2 mice) (**C**) The curvature of crypts in human tissue is plotted as Box plot for normal (one patient, n = 106, blue) and FAP (one patient, n = 100, orange) tissue revealing that in FAP tissue crypts are more highly curved. Statistical evaluation was performed using an unpaired t-test with Welch’s correction (assuming samples have unequal variances). ns = not significant, *p < 0.05, **p < 0.01, ***p < 0.001, ****p < 0.0001.

Differences in crypt packing could not be explained by differences in crypt diameter, prompting us to investigate whether crypts in precancerous mouse tissue had different shapes. To this end, crypt depth was measured by marking the top and bottom position of a crypt, and crypt length was measured by recording the length of a crypt lumen marked by Phalloidin (Fig. 4A). These measurements produced values similar to those previously reported for crypt length in mouse jejunum (Region 2) of approx. 70 μm with 16–17 cells/diameter^25^,^26^. Dividing crypt length by crypt depth produces an index of curvature (Suppl. Table 5), with an index of 1 indicating perfectly straight crypts and <1 indicating curved crypts. Occasionally indices >1 were introduced by minor discrepancies in measuring the top/bottom positions of crypts.

The most notable difference between WT and *Apc*^*Min*/+^ tissue was that in *Apc*^*Min*/+^ tissue, crypt shape was significantly more variable in regions 1, 4 and 5 (Suppl. Table 5). This was reflected by smaller minimum values indicating that there were more extremely curved crypts, particularly in Region 5 where several crypts in *Apc*^*Min*/+^ tissue produced values as low as 0.51, compared to a minimum score in WT crypts of 0.73 (Fig. 4B, Suppl. Figure S4A). The frequency distribution for curvature indices in the *Apc*^*Min*/+^ crypts (Suppl. Figure S4A) suggested that two populations of crypts exist in *Apc*^*Min*/+^ tissue: relatively straight crypts that are similar to those in WT tissue and others that are significantly more curved. We found that the highly curved crypts were longer by more than 30%, suggesting a positive correlation between crypt length and curvature.

In human FAP tissue crypts were also significantly more curved than in normal tissue, consistent with a greater number of curved crypts in FAP tissue (FAP 55% *vs.* normal 34.91% with curvature index ≤0.90) (Fig. 4C, Suppl. Figure S4B, Suppl. Table 6). Our high-resolution, three-dimensional imaging data show that the organisation of *Apc* heterozygous tissue is aberrant, with crypts more irregularly spaced and less straight. These changes could be responsible for the higher and more variable BSC and Z values recorded by μUS in this tissue.

## Discussion

The packing of crypts into regular arrays within the gut wall ensures a relatively uniform density of functional units. Although crypt depth and circumference vary along the stomach - rectum axis, crypts are shaped and organised consistently in all regions. Normally, crypts run relatively straight from the gut wall towards the lumen to produce regular packing.

Based on the idea that size and shape of crypts is dictated by the cells they contain we aimed to establish if features that were affected by even minor cellular changes produced by cancer–initiating mutations could be detected in tissue. To this end we compared quantitative μUS measurements from WT and *Apc*^*Min*/+^ mice and corresponding human patient tissue with high-resolution 3D optical data obtained from crypts in small and large intestine. Heterozygosity in *Apc* is predicted to produce several effects including decreased cell migration and potentially elevated proliferation through cytoskeletal changes and elevated availability of β-catenin, respectively^27^,^28^,^29^. Indeed, proteomic and genomic analysis of *Apc* heterozygous and homozygous mutant human and animal tissue reveal changes relating to apoptosis, responses to oxidative stress, differentiation, and Notch signaling^10^,^11^,^12^. Some alterations in tissue organisation in *Apc* heterozygous tissue has also been reported previously^30^,^31^,^32^,^33^. For instance, the broadening and upward extension of the proliferative zone that we detected in *Apc* heterozygous crypts (Suppl. Table 7) has previously been described. However, previous data was obtained in colonic (not small intestinal) crypts of FAP patients, or in *Apc*^*1638*/+^ mice, which are less tumour–prone than *Apc*^*Min*/+^ animals, and had also been treated with either 1,2-dimethylhydrazine or radiation^34^,^35^,^36^. Our three-dimensional approach permitted detection of previously indistinguishable changes in tissue architecture. Importantly, corresponding changes were also measurable by μUS. Although the parameters we measured by μUS are characteristics of bulk tissue, they reflect changes at the cellular level. This is consistent with our finding that the less regular structure of *Apc* heterozygous tissue is a bulk property.

The highly bent, curved crypts we found frequently in *Apc* heterozygous tissue from mouse and man can arise when cells are not distributed uniformly along the crypt axis. A number of possible mechanisms could contribute to this change, for example migration defects and changes in Wnt target genes^27^,^28^,^37^. Wnt signalling may be slightly increased in *Apc*^*Min*/+^ crypts, which could induce small local changes in proliferation^38^,^39^,^40^,^41^ (reviewed by^42^). Decreased migration due to cytoskeletal defects in *Apc* heterozygous cells could reduce movement of cells along the crypt axis. Together with increases in proliferation, aberrant migration could cause an uneven accumulation of cells and induce curvature in a crypt. A small increase in the number of mitotic cells was indeed detected in *Apc*^*Min*/+^ tissue (Suppl. Table 8) suggesting that both of these processes may operate. Unfortunately, the relatively small number of mitotic cells per crypt (6–8) makes it difficult to obtain statistically significant data about a possible bias in their distribution in the plane of the tissue, which could be used to identify such clusters.

Another factor that could contribute to crypt shape is the compliance of cells in crypts and surrounding tissue. Changes in the mechanical properties of tumour cells have been detected in some epithelial cancers^43^. Alterations in the regulation of cytoskeletal proteins are predicted to result from *Apc* heterozygosity and could render cells more compliant. The resulting ability of cells to assume more variable shapes could increase the variability of crypt shape. Alternatively, changes in the contractility of non-epithelial cells surrounding crypts could also contribute to aberrant crypt shapes. In the model systems we use here, all cells are heterozygous for *Apc* raising the possibility that non-epithelial cells contribute to the changes we recorded. Only directly measuring and comparing the mechanical properties of wild type and *Apc* mutant cells and tissues will reveal how these factors contribute to the architecture of crypts. A useful tool to distinguish between the mechanical properties of epithelial versus non-epithelial cells and their contribution to tissue structure may be organoids, which only contain epithelial cells. However, at this time it is not possible to manipulate the mechanical properties of the material that supports organoid growth, without also changing the concentration of extracellular matrix components that provide necessary biochemical factors.

Another significant difference we detected was less regular packing of crypts in *Apc* heterozygous mouse and human tissue as illustrated by the more variable distance between neighbouring crypts. These differences could not be explained by differences in crypt diameter but are likely linked to increased crypt curvature because curved regions of crypts occupy a larger cross-sectional area in the plane of the tissue. However, cause and effect are difficult to assign. It is easy to appreciate that a more curved crypt would at certain points be more distant from other crypts. In the small intestine, increased curvature could also be induced when crypts that are irregularly spaced are forced to bend to allow merging with contiguous villi.

Adding a quantitative modality to histological examination can improve the ability to detect relatively subtle tissue abnormalities *in situ*. Ultrasound is used successfully to detect tissue abnormalities clinically, usually at frequencies that permit deep tissue penetration but not the resolution to measure detailed changes in tissue structure. Using higher μUS frequencies increases resolution (approximately 100 μm in x-z for the 45.0 MHz transducer with aperture 1.50 mm and f# 4 we used). Although this resolution is not sufficient to measure properties of individual crypts (diameter ≥50 μm) it can detect changes in their packing and overall structure. This is particularly obvious in polyps where optical images reveal an increased density of epithelial cells and myofibroblasts (Fig. 1). Importantly, the increased *Z* in these areas allows detection of polyps before they reach a diameter of 300 μm (Fig. 1), much earlier than possible with current methods. BSC measures how strongly objects scatter μUS waves; it also reflects their concentration and/or size and relates to their spatial distribution^23^. In isolated cells, the most effective scatterers are nuclei and differences in their density and compressibility compared with other intracellular structures causes scattering^20^,^23^. In cell aggregates, the distribution of nuclei also affects scattering with more irregular spacing leading to increased BSC^23^. At the frequency we used, objects that scatter effectively are in the approximate size range 5–90 μm. This corresponds to cellular and tissue structures, enabling the detection of more irregularly packed crypts, cells, and nuclei in precancerous tissue. Indeed, the most robustly altered feature of precancerous tissue detected by optical imaging was an increase in the variability of almost all physical properties of crypts we examined.

The μUS signal was collected from the jejunum of 3–5 different age-matched littermate pairs at different regions along the intestinal axis that were free of palpable or visible polyps. Together with the extremely low frequency of aberrant crypt foci (ACFs) in the small intestine of *Apc*^*Min*/+^ mice, the increased variability in tissue structure reported by μUS suggests that it is not caused by locally increased values for BSC and Z but is a property of the entire tissue^30^,^31^,^32^,^33^. Indeed, examining the μUS data closely (Suppl. Figure S5) revealed that the increased values and variance for BSC measured by μUS were the result of many different points that are distributed widely throughout the tissue and represent a common feature of *Apc*^*Min*/+^ tissue in general, consistent with our optical data.

The ability to detect changes in precancerous tissue using μUS opens up the potential to identify precancerous changes in patients. Nonetheless, further development of the correlation between the quantitative parameters provided by μUS (harmonics, varying wavelength) and histological detail will be required to validate μUS as a useful complement to existing modalities for detecting early lesions in patients.

Another important question to be addressed through future validation is how fixation affects μUS measurements in intestinal tissue. We used fixed material so that optical data could be directly compared with μUS measurements. Although fixation can affect the absolute values of BSC and Z, relative differences between readings in different samples are maintained^44^. Comparing tissue samples prepared identically as we have done here yields relevant information about acoustic differences in tissue samples that can inform about pathological changes^44^,^45^,^46^. An important next step in developing μUS as a clinically useful tool for detecting early tissue abnormalities will be to establish exactly how fixation impacts on Z and BSC values in gut tissue. This requires a direct comparison of acoustic properties of non-fixed normal, pre-cancerous, and cancerous tissue from patients and in relevant animal models.

Ongoing development of μUS transducers that are sufficiently small to permit incorporation into devices that can be swallowed^47^,^48^ has the potential to greatly enhance our ability to investigate diseases of the small and large intestine. The ability to detect aberrant tissue reliably and in real time could report tissue changes over time, which would allow progression of disease to be monitored, allowing the stratifying of patients by differentiating stable from progressing tissue changes. It may also be applicable for reporting response to therapy and other clinical data e.g. surgical margins, without the need for biopsy.

## Materials and Methods

### Preparation of mouse tissue

All experiments involving animals were performed in accordance with UK Home Office approved guidelines and were approved by the Home Office Licensing committee (Project license PPL60/4172). For optical imaging, CL57BL/6 WT and *Apc*^*Min*/+^ male littermates aged 60–74 days were sacrificed by cervical dislocation and the entire intestine (small and large) was removed immediately. Tissue was washed and divided into proximal, medial and distal regions of the small and large intestines using percentage length from the gastro-duodenal and ileo-caecal junctions^49^ corresponding to six regions: small intestine: region 1, 10% = duodenum, region 2, 50% = jejunum, region 3, 90% = ileum; large intestine: region 4, 10% = caecum, region 5, 50% = transverse, region 6, 90% = distal (Suppl. Figure S1). A 5 mm long section from the middle of each of these six regions was immersed in cold fixative containing 4% paraformaldehyde at pH 7.4 overnight at 4 °C before processing for staining^50^.

Tissue for μUS scanning of the small intestine was removed immediately after cervical dislocation of at least three, of each CL57BL/6 WT or *Apc*^*Min*/+^ male and female littermates aged 60, 90 or 120 days. The intestine was flushed with cold PBS, tissue was cut longitudinally, pinned onto 3% agarose and fixed for 24 hours in 4% paraformaldehyde (PFA, pH 7.4) at 4 °C. After washing in degassed PBS at room temperature, it was re-pinned onto 1% agarose and covered with degassed PBS.

### Human tissue

Collection of tissue samples was approved by the Tayside Tissuebank subcommittee of the Local Research Ethics Committee with informed consent from all subjects. All tissue collection protocols were carried out in accordance with approved guidelines. Normal human sample was obtained from a surgical resection for hemi-colectomy. After specimen removal, macroscopically normal tissue, far removed from tumour–containing regions was excised by a Consultant Pathologist and divided into 5 mm long sections. Normal biopsies from one FAP patient were obtained during routine colonoscopy surveillance. All specimens were fixed with 4% paraformaldehyde at pH 7.4 and stored overnight at 4 °C before processing.

### Tissue staining for visualising F-actin and nuclei

Tissue samples were prepared for optical imaging of F-actin and nuclei as described previously^50^. BABB, which was used to clear and mount the tissue samples, causes significant shrinkage because it dehydrates tissue up to 28%^50^. This shrinkage has to be considered when comparing the absolute values of our measurements with those from studies using other tissue preparation methods^25^.

### Immunofluorescence

Tissue was dissected and stained as described previously, but fixed in methanol at −20 °C overnight^50^. Rabbit polyclonal antibodies against phosphorylated Histone H3 (Abcam ab5176) were used at a dilution of 1:500 and secondary antibody (goat anti-rabbit labelled with Alexa568, Molecular Probes A11036) was diluted 1:250. Samples were embedded in TDE as described previously^50^.

### Microscopy

Tissue was imaged with a Zeiss LSM 710 microscope using a 25x/0.8NA Zeiss objective and immersion oil with refractive index of 1.516. Multiphoton excitation was provided by a Coherent Chameleon Titanium Sapphire laser at 820 nm to simultaneously excite Hoechst and Alexa 568.

### Microscopy image analysis

Image visualization, 3-D rendering, and quantitative measurements were performed using Volocity tools (Volocity 5.5.1, Perkin-Elmer) as described in detail in the supplemental material. *Crypt depth* was recorded by scrolling through z-stacks to identify the top and bottom of each crypt and half height was defined as the midpoint between these points, where most measurements were made unless stated otherwise. *Crypt packing* was measured in the Z-stack at average half height in randomly selected fields of crypts using at least four different image stacks from at least two different mice.

### Microultrasound scanning

To obtain μUS images, a single-element focused piezocomposite transducer operating at a frequency of 45.0 MHz (λ = 34.2 μm) was positioned 5 mm above the tissue, corresponding to the focal distance of the transducer. (Technical details of the transducer are provided in the supplemental material). A LabVIEW (National Instruments, TX, USA) controlled scanning system was used to acquire the data. Using two stepping motors (Shot 602, Sigma Koki, Tokyo, Japan) mounted on an optical breadboard for stability, the transducer was moved linearly across the width of the sample (12 mm) in 17 μm steps and this was repeated at ten different positions (1 mm apart) along the intestinal axis. An exclusion parameter was applied to the scans to avoid artefacts caused by excessive curvature of the fixed tissue. Backscatter coefficient (BSC) and acoustic impedance, *Z*, in pulse-echo mode were calculated as described previously^22^,^51^,^52^ and in the supplemental material. Values for means from each scan were averaged from at least three scans for each sample set and plotted with standard deviations (Fig. 2). A quartz flat was used as a reference to allow the removal of contributions from system and transducer transfer functions, to calculate *Z*, to normalize BSC, and for calibration^22^,^53^.

### Statistical analysis

All statistical analyses were performed using GraphPad Prism 5.0a (GraphPad Software, Inc. CA, USA) for Mac OS. Specific statistical tests applied and relevant p-values are listed in the Figure legends.

## Additional Information

**How to cite this article**: Fatehullah, A. *et al.* Increased variability in *Apc*^*Min*/+^ intestinal tissue can be measured with microultrasound. *Sci. Rep.* **6**, 29570; doi: 10.1038/srep29570 (2016).

## Supplementary Material

Supplementary Information
