# Supplementary material for: Increased variability in ApcMin/+ intestinal tissue can be measured with microultrasound
Source: Sci Rep. 2016 Jul 13;6:29570. doi: 10.1038/srep29570 (PMC4942766; doi:10.1038/srep29570)
Supplement: Supplementary Information [file srep29570-s1.pdf]

Supplemental Material for:

Increased variability in *Apc*$^{Min/+}$ intestinal tissue can be measured with microultrasound

A. Fatehullah[1,3], S. Sharma[2,4], I.P. Newton[1], A.J. Langlands[1], H. Lay[2,6], S.A. Nelson[1,5], R.K. McMahon[1], N. McIlvenny[1], P.L. Appleton[1], S. Cochran[2,6], I.S. Näthke[1,*]

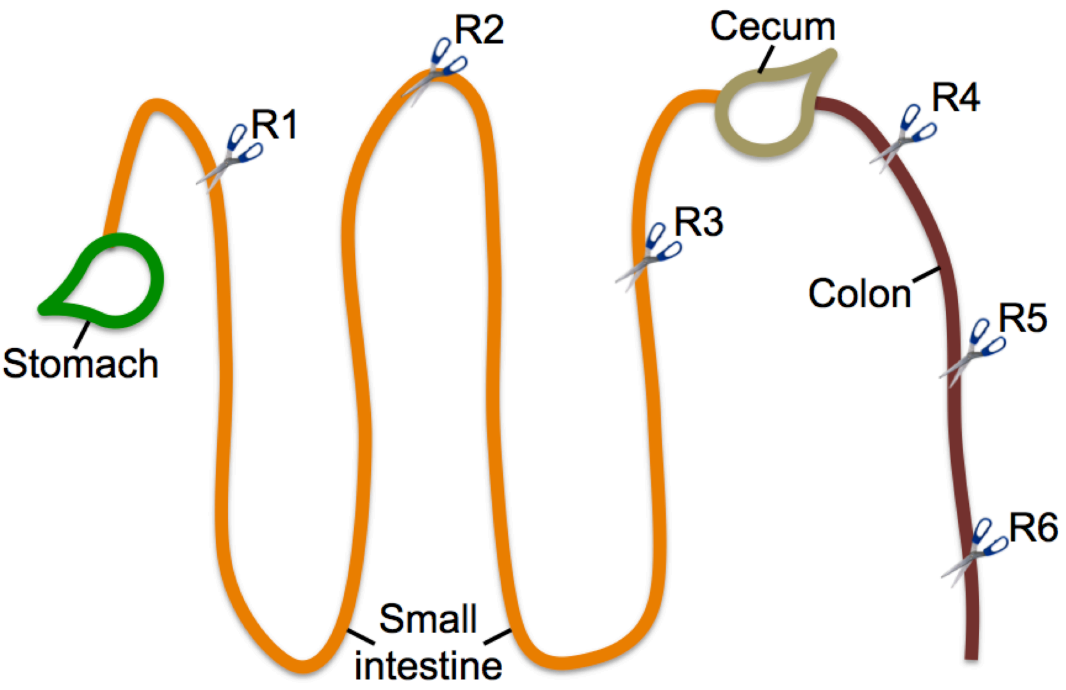

Supplemental Figure S1: Schematic of mouse intestine showing the positions of the regions (R1 – R6) along the stomach rectum axis that were used for collecting imaging and ultrasound data.

Supplemental Figure S2: Single B-scan of mouse intestine from an *Apc*$^{Min/+}$ mouse scanned with a 45MHz ultrasound probe. This is one of the 580 individual B-scans used to construct the three-dimensional image in Figure 1C. The high intensity signal at the underside of the tissue corresponds to the muscle layer (white arrowheads).

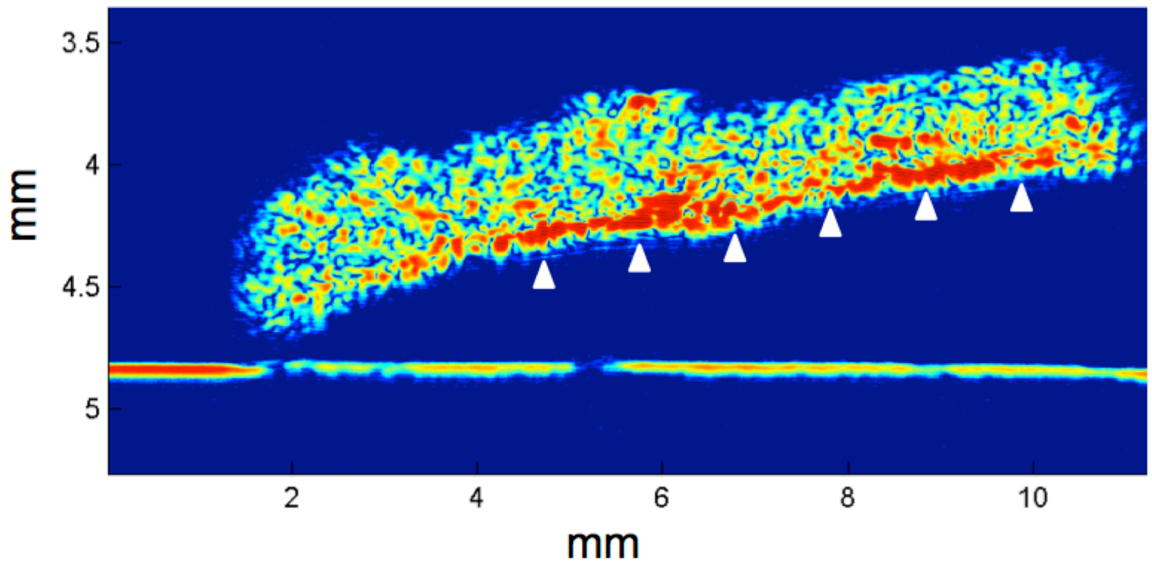

Supplemental Figure S3

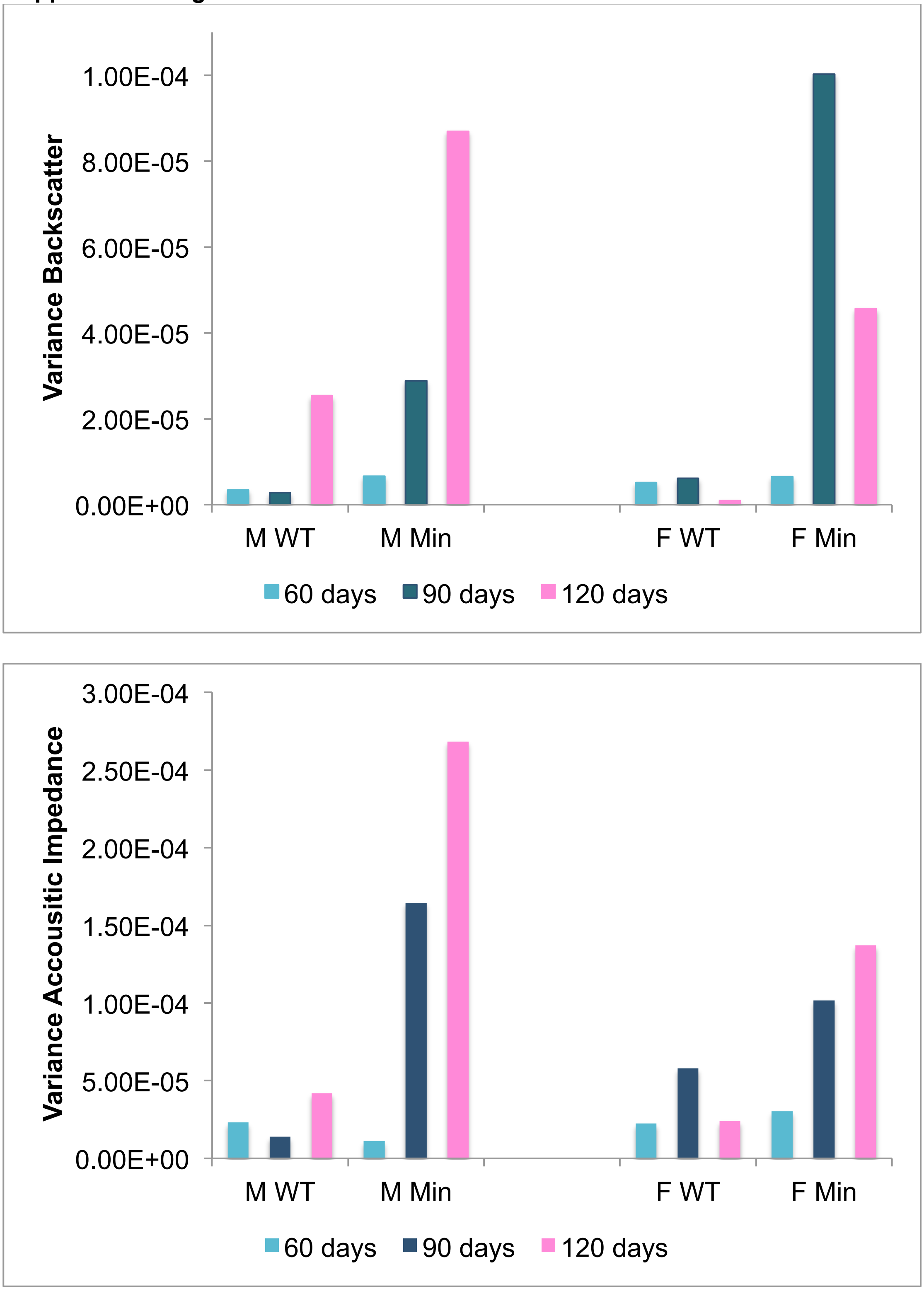

The variance in backscatter (top panel) and acoustic impedance (bottom panel) values were calculated for each tissue type (based on data shown in Figure 2) revealing increases in both measures in *Apc*$^{Min/+}$ tissue with age, consistent with increased variability in tissue structure. M = Male, F = Female, Min = Apc$^{Min/+,}$, WT = wild type.

Supplemental Table 1: To assess the significance of difference in the variance (Var) between Backscatter (BSC) and Accoustic Impedance (AI) of WT and *Apc*$^{Min/+}$ tissue presented in Figure1 and S1, t-tests were performed between the mean and standard deviation data sets for each sub-group and the calculated p-values are tabulated below. Lower p-values have been coloured a darker shade for clarity.

	60		90		120	
	F	M	F	M	F	M
BSC	0.0236	0.0863	0.0006	0.02	0.0034	0.0597
BSC var	0.0547	0.2953	0.0054	0.0954	0.0264	0.0639
AI	0.4037	0.0019	0.0004	0.0701	0.0316	0.0007
AI var	0.0294	0.0042	0.0019	0.1041	0.0946	0.0004

Supplemental Figure S4A

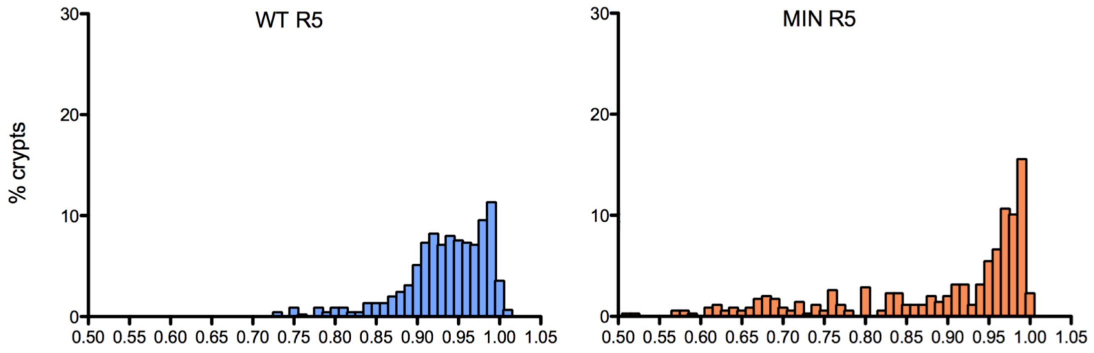

Supplemental Figure S4B

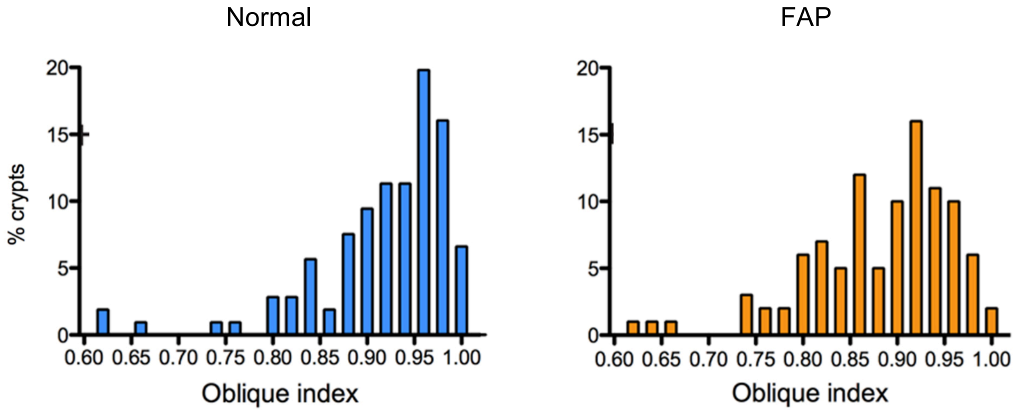

The curvature expressed as oblique index in Figure 2 is shown as histogram for wild type (WT, blue) and *Apc*$^{Min/+}$ (MIN, orange) mouse tissue from region 5 (A) and human tissue from normal and FAP tissue corresponding to region 3 (B). The histologically normal *Apc* heterozygous tissue had more severely curved crypts than wild type (mouse) or normal (human) tissue.

Supplemental Figure S5

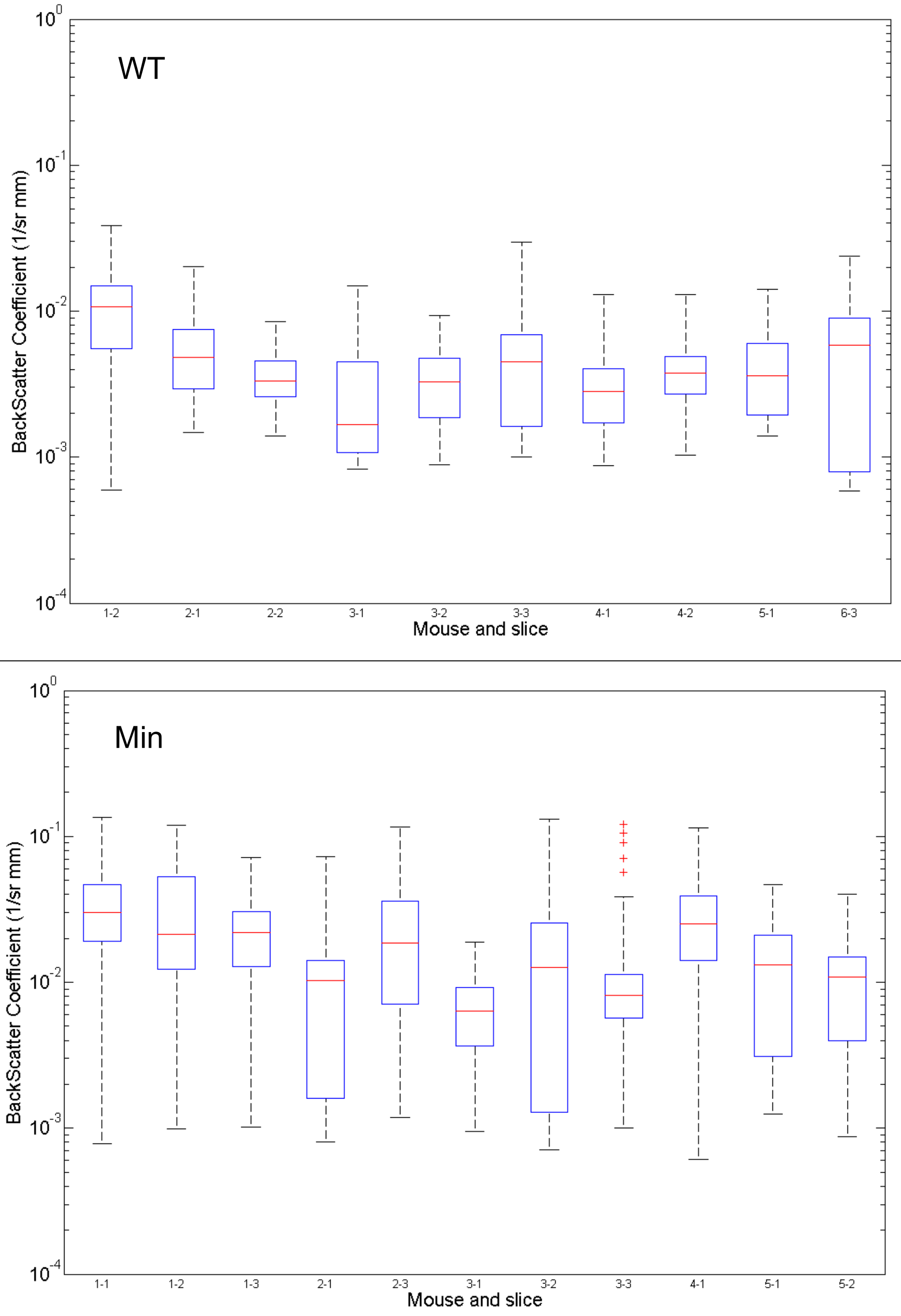

Backscatter data from individual µUS scans in female wild type (WT) and *Apc*$^{Min/+}$ (Min) animals aged 90 days were plotted to illustrate the distribution of values in each scan. Red lines indicate means, upper and lower box limits show 25% and 75% boundaries above and below the mean, and whiskers reflect maximum and minimum values, with outliers with values of more than 5 sigma plotted as red crosses. The expanded range of data distribution in the *Apc*$^{Min/+}$ tissue, with both broadening of the range containing 50% of the values closest to the mean and an increase in maximum and minimum values, shows a consistent broadening of backscatter variance across the breadth of the tissues and across all scans.

Tables summarising different quantitative crypt dimensions in the intestinal tract.

Supplemental Table 2: Crypt diameter [µm] in wild type (blue) and *Apc*$^{Min/+}$ (orange) crypts, in all six regions of the mouse intestine.

	R1	R1	R2	R2	R3	R3	R4	R4	R5	R5	R6	R6
Minimum	23.54	23.22	29.61	29.98	26.19	26.92	22.35	26.26	29.97	31.52	25.17	28.73
Maximum	47.20	45.30	58.60	54.15	40.47	41.93	40.47	38.36	68.50	59.93	51.02	42.97
**Mean**	**34.44**	**32.07**	**42.38**	**38.6**	**32.98**	**33.86**	**29.15**	**31.23**	**49.84**	**43.73**	**33.75**	**37.68**
Std. Deviation	5.07	5.53	5.80	4.96	3.42	3.23	4.11	3.35	10.05	5.17	5.72	3.54
p-value	0.7524		0.6201		0.8520		0.6957		0.7326		0.6437	
Coefficient of variation(%)	14.72	17.24	13.69	12.84	10.38	9.55	14.09	10.74	20.17	11.83	16.95	9.39
Skewness*	0.239	*0.553*	0.048	*0.797*	*0.537*	0.158	*0.835*	*0.761*	0.063	0.331	**1.038**	*-0.670*
Number of crypts	100	100	150	200	50	50	50	50	150	49	100	50
Number of mice	2	2	3	4	1	1	1	1	3	1	2	1

*Italics = moderately skewed   Bold = highly skewed

Supplemental Table 3: Regularity of crypt packing in wild type (blue) and *Apc*$^{Min/+}$ (orange) tissue, in all six regions was assessed by measuring the distance [µm] between neighbouring crypts. Analysing the distribution of the obtained values revealed that data in Apc$^{Min/+}$ tissue are not distributed normally and tend to be more highly skewed in regions 1-4. Skewness values below 0.5 reflect a uniform distribution, while values between 0.5 – 0.9 are slightly skewed, and values equal ≥ 1 are considered highly skewed.

	R1	R1	R2	R2	R3	R3	R4	R4	R5	R5	R6	R6
Minimum	19.52	22.18	25.92	23.41	20.14	26.69	20.06	27.36	30.14	23.95	19.99	16.19
Maximum	79.88	93.07	76.15	97.71	58.56	89.94	49.22	58.93	93.1	112.3	79.08	73.31
**Mean**	**42.79**	**45.01**	**48.41**	**46.39**	**39.56**	**47.57**	**34.39**	**37.97**	**56.11**	**57.22**	**38.77**	**40.15**
Std. Deviation	13.38	16.13	9.98	12.26	7.61	13.42	6.57	7.37	11.93	15.75	9.47	10.40
p-value Mean	0.2462		0.0629		0.0001		0.0059		0.408		0.2846	
p-value Variance	0.0424		0.0037		<0.0001		0.3803		0.0001		0.3066	
Skewness*	*0.603*	**1.086**	0.166	*0.659*	-0.055	*0.660*	0.358	*0.743*	*0.615*	*0.529*	*0.588*	0.457
Number of crypts	120	120	180	240	60	60	60	60	180	240	120	120
Number of mice	2	2	3	4	1	1	1	1	3	2	2	1

*Italics = slightly skewed   Bold = highly skewed

Supplemental Table 4: Inter-crypt distance and crypt diameter [µm] in normal (blue) and FAP (orange) human tissue. Tissue from one wild type and one FAP patient was used to obtain data from the indicated number of crypts.

**Human R3**	**Crypt distance**		**Crypt diameter**	
	**normal**	**FAP**	**normal**	**FAP**
**Mean**	**90.47**	**102.30**	**62.51**	**63.90**
Std. Deviation	23.40	28.91	9.67	9.45
Minimum	44.71	43.50	46.02	40.76
Median	89.34	80.60	59.88	62.49
Maximum	138.20	185.90	96.40	95.94
p-value Mean	<0.0001		0.2987	
p-value Variance	0.0011		0.8221	
Skewness	0.133	0.417	1.274	0.402
Number of crypts	240	240	106	100

Supplemental Table 5: Mean curvature index for all 6 regions of the intestine, wild type (blue) and *Apc*$^{Min/+}$ (orange) was measured as described in the materials and methods as the ratio of crypt height and length. Significance values were calculated using a two – tailed t test. Variance was calculated using the two - tailed F test. (ns) denotes non significant values. Either mean or variance or both is significantly different in all regions of mouse intestine and colon except R3.

	**Small Intestine**			**Colon**		
	**R1**	**R2**	**R3**	**R4**	**R5**	**R6**
**Mean**	**0.926**	**0.922**	**0.964**	**0.966**	**0.933**	**0.974**
Standard deviation	0.048	0.061	0.025	0.031	0.053	0.039
Number of crypts	150	597	200	200	450	150
Number of mice	2	5	2	2	5	3
**Mean**	**0.952**	**0.941**	**0.962**	**0.963**	**0.887**	**0.963**
Standard deviation	0.031	0.046	0.028	0.041	0.120	0.039
Number of crypts	150	649	200	200	349	100
Number of mice	3	5	2	2	2	2
p-value Mean	<0.0001	<0.0001	0.5354	0.4011	<0.0001	0.0344
p-value Variance	<0.0001	<0.0001	0.1685	<0.0001	<0.0001	0.8071

Supplemental Table 6: Crypt depth [µm], length [µm] and curvature index in human tissue in Region 3. Numbers from normal crypts are highlighted in blue and numbers from FAP crypts in orange. Tissue from one normal and one FAP patient was used to measure the indicated number of crypts. Although the mean values are not significantly different for crypt depth and length they are more variable in FAP tissue and span a larger range of values as indicated by higher maximum and lower minimum values. Importantly, FAP crypts are significantly more curved. The correlation between curvature and crypt depth is similar in normal and FAP tissue, but only in normal tissue is there a correlation between curvature and crypt length.

**Human R3**	**Crypt depth**		**Crypt length**		**Curvature Index**	
	**normal**	**FAP**	**normal**	**FAP**	**normal**	**FAP**
Minimum	72.00	64.00	82.17	81.10	0.626	0.614
Maximum	146.00	168.00	155.10	175.00	1.000	0.998
**Mean**	**106.30**	**105.50**	**116.00**	**119.10**	**0.918**	**0.882**
Std. Deviation	15.01	23.48	14.28	21.47	0.073	0.077
p-value Mean	0.1765		0.7425		0.0007	
Skewness	0.006	0.787	0.102	0.718	-1.870	-1.116
Correlation with curvature	0.379	0.628	0.284	-0.122	-	-
Number of crypts	106	100	106	100	106	100

Supplemental Table 7: Position of mitotic, PH3 positive cells relative to crypt depth [% of total depth measured from the crypt base], in wild type (WT, blue, n = 150 crypts) and Apc$^{Min/+}$ (orange, n = 150 crypts) crypts, in Region 2.

	**WT**	**MIN**
**Mean**	**55.03**	**53.64**
Std dev	18.55	20.21
Minimum	16.92	14.29
Maximum	98.67	110.5
Variation (%)	33.69	37.67
Number of crypts	150	150
Number of mice	3	3

Supplemental Table 8: Number of Phospho-Histone (PH3) positive mitotic cells (left) and number of cells in crypt circumference at half height (right) in wild type (blue) and *Apc*$^{Min/+}$ (orange) crypts, in Region 2.

PH3 positive cells	WT	MIN
**Mean**	**6.120**	**8.464**
Std dev	3.502	5.697
Max	18	25
Min	0	0
Skewness	0.6832	0.9521
Variation	57.23%	67.31%
Significance	0.0016 **	
Number of crypts	150	150
Number of mice	3	3

Circumferential cells	WT	MIN
**Mean**	**16.61**	**14.31**
Std dev	2.541	2.088
Max	25	23
Min	10	9
Skewness	0.2086	0.6734
Variation	15.30%	14.60%
Significance	<0.0001 ***	
Number of crypts	150	200
Number of mice	3	4

Supplemental Methods

Methods:

Transducer: The active element of the transducer is 1.50 mm in diameter. It is spherically focused and operates at a centre frequency of 45.0 MHz with an f-number of 2.85, providing a theoretical lateral resolution of 94.0 µm and an experimental lateral resolution of 107 µm at the focus of 4.25 mm. The experimental bandwidth of the transducer was measured to be 26.0 MHz. The beam geometry data were obtained using a phantom with 5 µm tungsten wires representing ideal line reflectors. Pulse transmission control and receive signal amplification was achieved with a commercial pulser-receiver (DPR 500, JSR Electronics, NY, USA) with the following settings: *Trigger*: internal; *Pulse repetition frequency:* 200 Hz; *Pulse energy:* 4µJ; *Damping:* 100 Ω; *Receiver Bandwidth: 5-300 MHz*; and *Receiver Gain*: 40 dB. The scan points were separated by 17.0 µm, i.e. approximately 1/6 of the beam width at the focus to achieve 3x spatial oversampling.

At each scan point, the radio frequency (RF) data was collected with a digital oscilloscope (HP 54810A, Agilent Technologies, Santa Clara, USA) at a sampling frequency of 1 GHz. A temporal average of 128 echoes was used to produce each A-scan (amplitude scan) to decrease noise. 9-bit data from the oscilloscope after averaging was transferred to the computer via a GPIB IEEE interface. All data processing of the ultrasound data was performed using MATLAB (Natick, MA, USA; www.mathworks.com).

The RF data were used to produce B-scan (brightness scan) images, and also to compute acoustic impedance (*Z*), attenuation coefficients (α') and BSC (BackScatter Coeffcients). Multiple 2-D B-scan images were 'stitched' using ImageJ (http://imagej.nih.gov/ij/index.html) to produce 3-

D ultrasound images for correlation with optical images (Figure 1). Images were segmented to remove biasing of the quantitative measurements by the muscle layer echo.

To calculate *Z*, it is necessary to know the amplitude of the transmitted ultrasound signal incident on the tissue. This can be calculated using a reference target (a quartz flat) with a known acoustic impedance imaged with the same settings as the tissue samples. The reflected amplitude, A_{ref}, was drawn from the maximum reflected voltage amplitude from the quartz optical flat. The reflectance, R, or the percentage of ultrasound energy reflected back to the receiver, was computed with

$$\boldsymbol{R = (Z_s - Z_0)/(Z_s + Z_0)} \qquad \textit{Equation 1}$$

where Z_s and Z_0 are the known acoustic impedances for an x-cut quartz flat of thickness 500 µm i.e. Z_s = 12.48 MRayl, and water, Z_o = 1.48 MRayl. The peak incident amplitude (PI) is then found from:

$$\boldsymbol{PI = A_{ref}/R} \qquad \textit{Equation 2}$$

where A_{ref} has been defined previously.

For each A-scan, the acoustic impedance of the tissue, Z_T, was then calculated using:

$$\boldsymbol{Z_T = -Z_0 \frac{(PI+PR)}{(PR-PI)}} \qquad \textit{Equation 3}$$

where PR is the voltage amplitude representing the peak reflected signal from each A-scan. The acoustic impedance was calculated for each A-scan over the entire tissue specimen then the mean, standard deviation and maximum values for each B-scan were calculated.

To calculate the backscatter coefficient (BSC), equation 4 was used, as shown in the literature. The unit of BSC is steradians per mm ($sr^{-1}mm^{-1}$).

$$\boldsymbol{BSC = \frac{R_q}{2\pi(1-cos\theta_T)} * \frac{\int_{t1}^{t2} |v_{tissue}|^2 dt}{\int_{-\infty}^{\infty} |v_{reference}|^2 dt} * \frac{2\alpha'}{(e^{-2az_1})(-e^{-2az_2})}} \qquad \textit{Equation 4}$$

α' is the intensity attenuation coefficient in exponential units, R_q is the intensity reflection coefficient of the water - quartz interface, V_{tissue} is the measured scatter signal and $V_{reference}$ is the signal measured from the quartz interface at the focal point of the transducer. θ_T is the half angle subtended by the transducer at the focus. z_1 and z_2 are the start and end depths of the tissue extents in the y-direction. The equation does not consider frequency dependent attenuation

effects and in effects computes the attenuation coefficient at the centre frequency of the transducer, i.e. 45.0 MHz.

To implement this equation in MATLAB, the averaged, gated RF echo signal was segmented to remove any biasing due to the muscle signal, squared and integrated over each B-scan. To remove system dependent effects, the previously obtained quartz reference signal was also squared and integrated along its maximum reflection A-scan and the integrated tissue signal was divided by this reference signal, then multiplied by the quartz reflectivity, R, calculated earlier.

To compensate for the loss of signal due to attenuation in the tissue, the attenuation factor is assumed to be constant and is integrated over the thickness of the tissue. To obtain the average attenuation in the tissue, the relative amplitude of the reflection from the 1% agar substrate under each slice of tissue was compared to the 1% agar reflection outside the physical extent of the tissue and the total attenuation calculated using equation 5.

$$\boldsymbol{attenuation\ (\alpha) = -\frac{20}{2l} * log_{10}\frac{A(x,y)}{A_0}\ \frac{dB}{mm}} \qquad \textit{Equation 5}$$

This was then converted to exponential units using equation 6.

$$\boldsymbol{\alpha' = \frac{2\alpha}{8.686}\ mm^{-1}} \qquad \textit{Equation 6}$$

Finally, to compensate for the angular fraction of the scattering sphere subtended by the transducer face, the half-angle of the transducer was calculated using equation 7.

$$\boldsymbol{\sin\theta_T = \frac{r}{D}} \qquad \textit{Equation 7}$$

where r is the radius of the transducer face and D is the focal distance.
